# Unveiling the hidden: acquired pediatric hypothyroidism

**DOI:** 10.3389/fendo.2025.1744280

**Published:** 2026-01-09

**Authors:** Mariacarolina Salerno, Donatella Capalbo, Mariella Valenzise, Raffaella Di Mase, Letteria Anna Morabito, Chiara Centonze, Malgorzata Gabriela Wasniewska

**Affiliations:** 1 Department of Translational Medical Sciences, Pediatric Endocrinology Unit, University of Naples Federico II, Naples, Italy; 2 Department of Mother and Child, Pediatric Endocrinology Unit, University Hospital Federico II, Endo-ERN Center for Rare Endocrine Conditions, Naples, Italy; 3 Clinical Research Center DEMeTra, Department of Translational Medicine, University of Naples Federico II, Naples, Italy; 4 Department of Mother and Child, Pediatric Unit, University Hospital, Endo-ERN Center for Rare Endocrine Conditions, Messina, Italy; 5 Department of Human Pathology of Adulthood and Childhood, Pediatric Unit, University of Messina, Messina, Italy, 6Medical Affairs, Merck Serono SpA, Rome, Italy; 6 Medical Affairs, Merck Serono SpA, Rome, Italy

**Keywords:** acquired hypothyroidism, central hypothyroidism, Hashimoto thyroiditis, primary hypothyroidism, subclinical hypothyroidism

## Abstract

Acquired hypothyroidism is the most common thyroid disorder in children, with Hashimoto thyroiditis representing the leading cause in iodine-sufficient regions. Children and adolescents may present with a broad spectrum of clinical signs and symptoms, ranging in severity. Unusual or atypical presentations can complicate the differential diagnosis, potentially resulting in delays in both diagnosis and treatment, which may significantly impact growth and developmental outcomes. This review presents four real-life pediatric cases that illustrate both typical and atypical manifestations of acquired hypothyroidism. These cases are accompanied by a comprehensive overview of the condition’s etiology, clinical presentation, diagnostic strategies, and management approaches in the pediatric population. The aim of this review is to summarize current knowledge on acquired hypothyroidism in children. By presenting real-world clinical cases, this review highlights key elements that can assist pediatricians in identifying and managing pediatric patients affected by this condition.

## Introduction

Hypothyroidism is a common condition characterized by insufficient production of thyroid hormone (TH). TH plays a critical role in regulating the metabolic activity of nearly all tissues and organs (1) and is essential for neurodevelopment, cardiac function, skeletal growth, gastrointestinal motility, and overall metabolic homeostasis. TH deficiency results in a wide range of clinical manifestations with severity varying according to the extent and duration of the hormone insufficiency (2, 3).

Overt hypothyroidism is defined by thyroid-stimulating hormone (TSH) levels that exceed the upper limit of the reference range associated with low levels of free thyroxine (FT4), whereas in subclinical hypothyroidism (SH), TSH levels are slightly increased, but FT4 concentrations remain within the normal reference range (4–6). The overall prevalence of hypothyroidism varies depending on nutritional iodine levels and may affect up to 12% of the general population (3, 5). However, a meta-analysis investigating the prevalence of undiagnosed hypothyroidism across European countries revealed that approximately another 5% of cases remain undiagnosed, mostly representing the subclinical form of the disease (6).

Hypothyroidism can be either congenital or acquired. While congenital hypothyroidism is routinely detected through newborn screening in several countries, acquired hypothyroidism can occur at any age and may be more challenging to identify (7, 8). Among children and adolescents, acquired hypothyroidism is more prevalent, affecting 1 in every 740 individuals, compared to congenital hypothyroidism, which occurs in 1 to 4,000 live births (9, 10).

Overall, acquired hypothyroidism can be classified as either primary, originating in the thyroid gland and presenting as overt or subclinical hypothyroidism, or central, resulting from dysfunction in the pituitary or hypothalamus (3). Autoimmune thyroiditis (AIT), also known as Hashimoto’s thyroiditis (HT), is the leading cause of acquired hypothyroidism in children in areas with adequate dietary iodine intake. Other common causes of acquired hypothyroidism include iodine deficiency, medications, and other iatrogenic factors (8).

Classical signs of overt hypothyroidism in children and adolescents include thyroid gland enlargement (goiter), impaired linear growth, delayed or arrested puberty, excessive weight gain, difficulty concentrating, cold intolerance, constipation, and myxedema of the face and extremities (11–13). However, atypical and non-specific features may also occur, and the disease presentation can be subtle (14, 15). As a result, achieving a timely diagnosis of acquired hypothyroidism in children is often challenging. Delays in appropriate diagnosis and treatment can impair growth and cognitive development, potentially leading to serious consequences for the child (16, 17).

The aim of this review is to summarize current knowledge on acquired hypothyroidism in children. By presenting real-world clinical cases, this review highlights key elements that can assist pediatricians in identifying and managing pediatric patients affected by this condition.

## Clinical cases

### Patient 1

A 13-year-old girl was referred to the pediatric endocrinology clinic for short stature and delayed puberty. She was born preterm (gestational age 33 weeks) and was small for gestational age, with a birth weight of 1000 g (-2.5 SDS) and a birth length of 33 cm (-3.6 SDS). However, she demonstrated satisfactory catch-up growth in the early life and maintained a regular growth trajectory until the age of nine years. In the subsequent three years, she experienced growth arrest with a total height increase of only 0.1 cm. Clinical examination revealed severe short stature (height 132.8 cm, -3.7 DS), weight excess (body mass index, BMI 21.7 kg/m^2^) and absence of pubertal development. Biochemical examination documented increased concentrations of creatinine (1.3 mg/dl) and urea nitrogen (53 mg/dl). Testing of thyroid function revealed severe hypothyroidism due to HT, characterized by high TSH levels (236.6 mIU/l, n.v. 0.3-4.2 mIU/l), low FT4 (0.25 ng/dl, n.v. 0.9-1.7 ng/dl), and high titers of anti-thyroglobulin (TG-Ab) and anti-thyroperoxidase antibodies (TPO-Ab) (>3000 and >1200 UI/ml, respectively). Karyotype and serum levels of other pituitary hormones were normal. Ultrasound evaluation of the thyroid revealed small, atrophic gland with hypoechogenic micronodules. Levothyroxine (LT4) treatment was promptly started (1.9 mcg/kg/day). After three weeks, a significant improvement in thyroid and renal functions was observed (FT4: 1.3 ng/dl, urea nitrogen: 38 mg/dl, creatinine: 0.9 mg/dl). Over the following months, the patient exhibited regular pubertal progression, a reduction in BMI as well as an improvement in school performance. Additionally, a significant catch-up growth was observed, although final height remained compromised at –2.5 SDS.

### Patient 2

A 13.5-year-old boy was referred to the pediatric endocrinology clinic due to obesity. He had experienced a rapid weight gain of 10 kg over of the previous three months, which was not associated to a significant increase in caloric intake. Additionally, he reported severe fatigue, excessive sleepiness, and chronic constipation. A significant decline in school performance was noted during the same period. Family history was positive for HT. On physical examination, his weight was 67 kg, with a BMI of 29.8 kg/m² (+2.07 SDS), his height was appropriate for his genetic target, and pubertal development was complete. Relevant findings included bradycardia, pallor, facial puffiness, dry skin, and non-pitting edema of the limbs. No thyroid enlargement was observed. Initial laboratory results showed marked increase in serum creatine kinase (CPK) levels (1020 U/L, n.v. 30–200 U/L). Moreover, anemia (hemoglobin 9 gr/dl), and moderate increase in total cholesterol (274 mg/dL) and triglycerides (295 mg/dL) were detected. Cardiac evaluation identified a minimal pericardial effusion, with otherwise normal cardiac structure and function. Electrocardiogram confirmed bradycardia, without additional abnormalities. Mild hepatomegaly was also noted. The evaluation of thyroid function revealed overt hypothyroidism characterized by increased TSH levels (>150 mIU/l) with markedly reduced FT4 (0.12 ng/dl, n.v. 0.75-1.7 ng/dl) and FT3 (0.6 pg/ml, n.v. 0.5-4.9 pg/ml) levels. TG-Ab and TPO-Ab were markedly increased (103 and >1300 UI/ml, respectively) and ultrasound showed normal thyroid volume with a heterogeneous pattern and pseudonodular appearance of the gland, leading to a definitive diagnosis of HT. LT4 therapy was started at 50 mcg/day and titrated up to 150 mcg/day (2.2 mcg/kg/day). Within six weeks, thyroid function normalized. After four weeks of treatment, the pericardial effusion resolved completely, and CPK, cholesterol, and triglyceride levels returned to normal levels. Over the following 10 months, the patient lost 9 kg, myxedema completely resolved, and a marked improvement in energy levels and daily functioning was reported.

### Patient 3

A 7.5-year-old boy was referred to the pediatric endocrinology clinic for short stature. Physical examination confirmed short stature (height 113.4 cm, -2.15 SDS), below the genetic target. Weight (19.9 kg) and BMI (15.5 kg/m^2^) were within the normal range for age. Bone age was delayed by 2.5 years. A progressive reduction in linear growth velocity was reported between the ages of 6 and 7 years. Laboratory tests showed slightly reduced FT4 levels (0.7 ng/dl, n.v. 0.75-1.7 ng/dl), with TSH levels within the normal range (2.1 mIU/l), thus suggesting central hypothyroidism. Further evaluation of pituitary function showed deficiencies in both adrenocorticotropic hormone (ACTH), with low basal (36 ng/ml) and stimulated (125 ng/ml) cortisol levels, and growth hormone (GH), with peak GH levels of 1.73 ng/ml after arginine and 1.56 ng/ml after glucagon stimulation. Replacement therapy was initiated, starting with hydrocortisone and followed by LT4, which normalized both adrenal and thyroid function. After 2 months, at the age of 7.7 years, the patient developed polyuria and polydipsia, leading to a diagnosis of diabetes insipidus. Vasopressin therapy was added to the treatment regimen. Brain MRI showed an intrasellar and suprasellar craniopharyngioma, which was histologically confirmed as adamantinomatous craniopharyngioma. The lesion was completely removed via transsphenoidal intranasal surgery. Six months after surgery, recombinant GH therapy was started, followed by catch-up growth.

### Patient 4

A 3-year-old girl was referred to the pediatric endocrinology clinic for recurrent pericarditis, fatigue, and muscle weakness. These symptoms were accompanied by recent onset of weight gain, swelling of the face, and chronic constipation. Family history was notable for HT, Graves’ disease, and hypercholesterolemia. Her height (93.2 cm, -0.53 SDS) and weight (14.6 kg) were normal for age and sex. She presented with pale and round face and a normal thyroid size, without palpable nodules or regional lymphadenopathy. The abdomen was distended, but soft on palpation. Hypertrophy of both calf muscles and non-pitting edema of the limb were noted, and blood pressure (120/60 mmHg) was high for age (>95^th^ centile). Cardiac evaluation showed pericardial thickening without hemodynamically significant dissection. Laboratory findings revealed markedly increased triglycerides and cholesterol levels, along with significantly elevated liver transaminases. Several causes of pericardial effusion and hypertransaminasemia, including infections, liver dysfunction, malabsorption, autoimmunity, and myopathies, were excluded. Evaluation of the thyroid function revealed overt primary hypothyroidism characterized by elevated TSH levels (536 mUI/l, n.v. 0.3-4.2 mIU/L), with decreased FT4 concentration (0.28 ng/dl, n.v. 0.9-1.7 ng/dl) and elevated TPO-Ab (4800 UI/mL), consistent with a diagnosis of HT. Ultrasonography revealed a mild enlargement of the thyroid gland volume, with increased vascularity. Initiation of LT4 therapy led to a progressive normalization of thyroid function and liver enzymes, as well as gradual resolution of pericardial effusion.

### Etiology and epidemiology acquired primary hypothyroidism

Acquired primary hypothyroidism in children arises from a wide range of underlying conditions. AIT represents the most prevalent cause of acquired hypothyroidism in adults and children and typically manifests as HT (3, 8, 18). In children, HT prevalence is estimated to be between 1% and 3%, with a peak incidence during early-to-mid-adolescence and a strong female predominance (11, 19, 20). HT is driven by a complex interplay between genetic susceptibility, environmental, and epigenetic factors, leading to immune tolerance breakdown. Pathogenetic pathways have not yet been fully elucidated, but both humoral and cytotoxic immune responses are involved. Circulating autoantibodies, mainly against TPO and TG, are present in most patients and appear to be predictive of the disease, having been hypothesized as some of the primary immune events leading to its development. Moreover, the interplay between autoreactive T and B lymphocytes, cytokine-mediated inflammation, antibody-dependent cytotoxicity, and apoptotic mechanisms drives progressive thyrocyte destruction and glandular atrophy (21).

Several studies have attempted to clarify the complexity of the genetic factors predisposing individuals to HT (22, 23). Among the several identified genes, those encoding the human leukocyte antigen (HLA) system, including *HLA-DR3*, *HLA-DR4*, and *HLA-DR5*, have been reported to be associated with HT. Moreover, mutations in the *CTLA-4*, *PD1*, *PTPN22*, *CD14*, *CD40*, and *IL2R* genes, as well as in thyroid-specific genes such as those encoding the TSH receptor and thyroglobulin, have been linked to HT susceptibility (21, 23–25).

The prevalence of HT is particularly increased in children with Turner syndrome (TS), Down syndrome (DS), and Klinefelter syndrome (KS), ranging between 10-21%, 13-34%, and 5.4-10%, respectively (26, 27). Moreover, HT is frequently associated with other autoimmune diseases; for instance, a prevalence of 23% has been reported in children with type 1 diabetes (28), 13% in those with juvenile idiopathic arthritis (29), and 10% in children with celiac disease (30). HT may also occur in the context of autoimmune polyendocrine syndromes, a group of clinical conditions characterized by functional impairment of multiple endocrine glands due to loss of immune tolerance (31, 32).

Atrophic autoimmune thyroiditis (AAT) is a rare variant of autoimmune thyroiditis, characterized by progressive thyroid atrophy and fibrosis without the typical enlargement seen in other forms of HT. AAT leads to irreversible primary hypothyroidism, requiring lifelong hormone replacement therapy, often representing the end stage of HT (15, 33). Similar to HT, the etiology of AAT remains unclear and involves a complex interplay of genetic and environmental factors.

Besides AITs, iodine deficiency significantly contributes to the global burden of acquired hypothyroidism in children (8). Iodine represents a critical substrate for TH synthesis; therefore, adequate iodine intake is crucial for normal thyroid function, brain development, growth, and metabolism. Iodine requirements change throughout life, being higher during rapid growth in childhood and adolescence (34–36). While iodine deficiency is a worldwide issue, its prevalence varies by region and is influenced by different factors, including local dietary iodine intake and the effectiveness of public health iodine supplementation programs (5, 35, 37). Salt iodization programs have significantly improved iodine intake in several iodine-deficient countries, reducing the prevalence of acquired hypothyroidism among children (38, 39). However, even in traditionally iodine-sufficient areas such as the USA and parts of Europe, recent declines in iodine nutrition levels have raised concerns due to shifts in dietary habits and food production practices (35, 40). Notably, an increase in cases of HT has been reported following prophylaxis with excessive doses of iodized salt (41).

Various other environmental factors may also contribute to the development of hypothyroidism in children. For instance, exposure to endocrine-disrupting chemicals (EDCs), including industrial pollutants and pesticides, can interfere with thyroid function at any level of the hypothalamic–pituitary–thyroid axis, including thyroid hormones synthesis, release, transport, metabolism, and action on target tissues (42, 43).

Medical interventions can also impair thyroid function in children. The most common iatrogenic causes of acquired hypothyroidism include thyroidectomy (44), radioactive iodine (RAI) therapy, and external radiation for treating malignancies (45–47). Additionally, hypothyroidism may result from the administration of medications that interfere with TH production, metabolism, or TSH secretion (44). For instance, lithium, commonly used in psychiatric conditions in adolescents, generates thyroid abnormalities by interfering with TH production and release (48); amiodarone, a potent antiarrhythmic agent, may lead to thyroid dysfunction by interfering with thyroid hormone production and metabolism, or through direct cytotoxic effects on the thyroid gland (49); and glucocorticoids may interfere with TSH production by directly affecting thyrotropin-releasing hormone (TRH) in the hypothalamus (50). Finally, antiepileptic drugs and immune-checkpoint inhibitors can also cause thyroid dysfunction of variable degree. Overall, medication-induced thyroid dysfunction is often reversible after treatment is discontinued (51, 52).

### Acquired central hypothyroidism

Central hypothyroidism (CeH) is a rare disorder characterized by inadequate TSH stimulation of an otherwise normal thyroid gland, due to anatomical or functional defects of the pituitary (secondary hypothyroidism) or the hypothalamus (tertiary hypothyroidism), resulting in varying impairments in TSH secretion (53, 54). Thus, in contrast to primary hypothyroidism, CeH is biochemically characterized by low serum FT4 in combination with low, normal, or mildly elevated TSH concentrations. Notably, elevated TSH levels in CeH may reflect the presence of qualitatively defective TSH isoforms, which retain immunoreactivity but exhibit reduced biological activity, thereby failing to adequately stimulate thyroid function (55).

CeH is less common than primary hypothyroidism and is often associated with deficiencies in other pituitary hormones, including GH, gonadotropins, and ACTH (53, 54). The overall prevalence is estimated to range from 1 in 16,000 to about 1 in 100,000 in the general population (53, 54).

Acquired CeH may arise from several different conditions, including tumors, infectious or inflammatory diseases, traumatic brain damage, pituitary adenomas, drugs, and radiation therapies, which involve the hypothalamic-pituitary region (55, 56). In particular, craniopharyngioma, a rare pediatric tumor, is one of the most common causes of acquired CeH in children, due to its proximity to the hypothalamic-pituitary axis (57). Indeed, TSH deficiency has been reported in 37 to 98% of children and adolescents with craniopharyngioma and can lead to significant long-term endocrine morbidity, especially when diagnosis is delayed (57, 58).

## Clinical presentations

### Acquired primary hypothyroidism

Primary acquired hypothyroidism in children and adolescents presents with a wide spectrum of clinical manifestations, whose severity varies according to the degree of thyroid dysfunction. In children with HT, thyroid function may range from euthyroidism to subclinical or overt hypothyroidism, and occasionally even hyperthyroidism, depending on the stage of the disease (59).

Overall, symptoms may not be evident at the onset of the disease, particularly in euthyroid or SH forms, and may develop over time. Notably, up to 80% of affected children may be asymptomatic at diagnosis (8, 60), and the prevalence of SH has been estimated at 1.7% among adolescents and 2.9% among children (61).

Signs and symptoms associated to acquired primary hypothyroidism can be typical or atypical (**Table 1**).

**Table 1:** Summary of the main typical and atypical signs and symptoms of acquired primary hypothyroidism in children and adolescents.

Typical signs and symptoms	Atypical signs and symptoms
Goiter	Renal function impairment
Growth delay/arrest	Muscular weakness/myalgia
Short stature	Joint pain
Delayed bone age	Rhabdomyolysis
Puberty delay/arrest	Mild hypertension
Menstrual irregularities	Pericardial effusion
Weight gain	Anemia
Bradycardia	Peripheral precocious puberty
Cold intolerance	Hypertrichosis
Fatigue	
Lethargy	
Dry skin	
Hair loss	
Constipation	
Impaired school performance	
Cognitive dysfunction	
Myxedema	

#### Typical signs/symptoms

One of the most common presenting signs of acquired hypothyroidism is an enlarged thyroid gland (goiter), particularly in children affected by iodine deficiency or HT (20, 62–64). Goiter typically arises as a compensatory response of thyroid follicular cells to reduced TH production and increased TSH levels. In HT, goiter formation is further driven by lymphocytic infiltration and thyroglobulin accumulation, which lead to inflammation and progressive fibrosis of the thyroid tissue (60, 64). Goiter is estimated to occur in approximately 70% of children with HT (60, 64), whereas the prevalence of goiter due to iodine deficiency varies significantly across geographic regions, reflecting differences in dietary iodine intake and public health interventions (35). Goiter may present with either a diffuse uniform enlargement of the thyroid gland or as a multinodular goiter. In severe cases, the increased glandular volume can exert compressive effects on adjacent structures, potentially leading to symptoms such as dysphagia, dyspnea, hoarseness, or a sensation of cervical tightness (64, 65). When a goiter is associated with one or more nodules, further diagnostic evaluation is warranted to exclude the presence of a neoplastic lesion, particularly when the nodule is solitary (64, 65).

In some cases, poor linear growth and delayed skeletal maturation are the initial findings of acquired hypothyroidism (66), as observed in the case of patient 1. Indeed, TH is responsible for chondrocyte development and growth within the growth plates, and for the stimulation of bone formation and resorption by promoting osteoblast proliferation and type 1 collagen expression (67, 68). Different studies found that over 70% of children with hypothyroidism experienced growth arrest or delay, with a significant proportion presenting with short stature (69). The degree of growth retardation has been shown to correlate with the severity of hypothyroidism at the time of diagnosis (70).

Delayed puberty, as observed in the case of patient 1, is another common feature in adolescents, resulting from the disruption of the interactions between the hypothalamic-pituitary-thyroid and hypothalamic-pituitary-gonadal axes. In particular, TH plays an important role in providing feedback to the hypothalamus, which is responsible for the release of the gonadotropin-releasing hormone (GnRH) (71, 72). Overt hypothyroidism can often be associated with excessive and rapid weight gain, as observed in the case of patient 2. TH is indeed involved in the regulation of basal metabolic rate, lipid and glucose metabolism, and fat oxidation (66, 73). Notably, AIT has been reported in 12% of obese children (74). Moreover, at the time of AIT diagnosis, hypothyroidism is more frequently detected in obese subjects than in non-obese patients (75). However, it is important to emphasize that obese children may often present with SH that is not associated with autoimmune thyroiditis or with other clinical signs or symptoms of overt hypothyroidism. Indeed, SH is observed more commonly in obese children, with a reported prevalence ranging from approximately 7% to 23% (76, 77), compared to a prevalence of 1.7% to 2% in the general pediatric population. Therefore, it has been suggested that, in obese children, SH may reflect an adaptive mechanism aimed at enhancing energy expenditure, resulting in increased TSH concentrations, although the underlying mechanisms remain unclear (78). Moreover, hypothyroidism can be associated with dyslipidemia, as observed in the cases of patients 2 and 4. Indeed, low TH levels influence both low-density lipoprotein (LDL) receptors in the liver, leading to decreased LDL catabolism, which regulates cholesterol levels in the blood, and the function of hepatic triglyceride lipase, which removes triglycerides from the blood (66).

TH plays a pivotal role in brain development and function (79). Children with overt hypothyroidism may develop cognitive dysfunction of varying severity, ranging from impaired memory, attention, and school performance (80, 81), as observed in the case of patient 2, to more severe cognitive decline.

Additionally, other clinical manifestations of acquired hypothyroidism can include chronic constipation, as seen in patients 2 and 4, as well as fatigue, sleepiness, cold intolerance, dry skin, which was noted in patient 2, and hair loss.

Chronic constipation may primarily result from reduced gastrointestinal motility, as low TH levels impair smooth muscle contraction, thereby slowing intestinal transit (79, 82). Fatigue, sleepiness, and cold intolerance are likely related to decreased metabolism and thermogenesis, caused by low TH concentrations (8, 83). Although the pathophysiological mechanisms underlying dry skin and hair loss remain not fully elucidated, TH is believed to play a key role in hair follicle biology by regulating the proliferation and differentiation of epidermal appendage cells. Furthermore, TH contributes to skin homeostasis by promoting keratinocyte proliferation and maintaining epidermal barrier integrity, which may account for the cutaneous manifestations observed in hypothyroid patients (84, 85).

In severe hypothyroidism, a rare clinical manifestation is myxedema, characterized by diffuse thickening and hardening of the skin due to increased deposition of connective tissue components, particularly glycosaminoglycans (86). This accumulation leads to fluid retention and swelling of the subcutaneous tissues, resulting in non-pitting edema, typically localized to the periorbital region, pretibial areas, and the hands and feet, as observed for patients 2 and 4. Consequently, facial appearance may change, exhibiting swollen lips, a broad nose, macroglossia, and puffy droopy eyelids (87, 88). If left untreated, myxedema can progress into a myxedema coma, a life-threatening condition characterized by hypothermia, altered mental status, bradycardia, decreased cardiac contractility, hypotension, hypoventilation, and metabolic alterations (5), which can be fatal in up to 60% of cases. However, it remains a rare occurrence, with only a limited number of cases having been reported in children so far (89).

Notably, TH exerts several relevant effects on the cardiovascular system (CV), regulating the expression of key cardiomyocyte-specific genes, and thus affecting heart contractile ability and electrophysiological activity (90, 91). Moreover, TH stimulates nitric oxide (NO) production, which is responsible for maintaining endothelial function and vasodilation (92). Consequently, hypothyroidism can lead to bradycardia (93–95), as observed in the case of patient 2, decreased cardiac output and left ventricular function, along with increased systemic vascular resistance (90, 92).

#### Atypical signs/symptoms

Many unusual signs and symptoms may be associated with overt acquired hypothyroidism (**Table 1**). Although not common, awareness of these signs is important, especially in children with a personal or family history that puts them at risk of developing hypothyroidism.

Among them, unusual CV signs include, mild hypertension and pericardial effusion (96–99), as seen for patient 4. Although rare, pericardial effusion leading to cardiac tamponade may occur in children with acquired hypothyroidism, as the accumulation of pericardial fluid has the potential to impair cardiac function (100). The pathophysiological mechanisms linking hypothyroidism to pericardial effusion are not fully understood; however, it has been proposed that low TH levels promote vascular permeability and reduce lymphatic drainage, resulting in fluid accumulation within the pericardial cavity (101).

TH also plays a critical role in multiple aspects of skeletal muscle physiology. Consequently, hypothyroidism may lead to alterations in muscle fiber composition, impaired oxidative mitochondrial metabolism, disrupted glycogen synthesis, and reduced cellular energy availability. These changes contribute to muscle weakness and increase susceptibility to muscle injury associated with elevated CPK levels (102), as observed in patient 2. In more severe cases, rhabdomyolysis can occur, eventually leading to multiorgan dysfunction (103, 104).

Although rare, impaired glomerular function and free water clearance with hyponatremia have been reported in patients with acquired hypothyroidism, as TH influences kidney development and function. Indeed, decreased TH levels may result in thickening of the glomerular basal membrane and expansion of the mesangial matrix (105–107). A strong correlation between the severity of hypothyroidism and increased creatinine levels has been reported, as reduced renal flow and glomerular filtration disrupt renal hemodynamics (106). In addition, kidney dysfunction can also be a consequence of myopathy and rhabdomyolysis, secondary to hypothyroidism (108).

Finally, another uncommon manifestation of hypothyroidism is anemia (109–112), as observed in the case of patient 2. Indeed, TH influences the production of erythrocyte precursors from the bone marrow by regulating oxygen levels, and directly affects their growth due to the presence of TSH receptors. Additionally, TH is involved in the production of hemoglobin protein chains (111).

Very rarely, children with acquired hypothyroidism may manifest peripheral precocious puberty, as occurs in Van Wyk-Grumbach syndrome (VWGS) (113). Manifestations include genital bleeding, cystic ovaries, breast enlargement, headaches, and delayed bone age in girls and macroorchidism without virilization in boys (114). In this condition, high TSH concentrations promote the expression of the FSH receptor in the gonads and act as “FSH-like” molecule on these receptors due to shared structural components, thus inducing precocious puberty in the absence of LH effects (71, 115, 116).

### Acquired central hypothyroidism

Typical signs and symptoms in patients with CeH are often nonspecific and are usually milder compared to those of primary hypothyroidism. It has been proposed that the potential residual activity of the pituitary gland producing TSH and TSH receptor activity might contribute to the lesser severity of nonspecific symptoms in CeH (117). In most cases, TSH deficiency is combined with multiple other hormone deficiencies, and additional signs and symptoms depend on the type and severity of pituitary dysfunction (118).

## Diagnosis and management

**Figure 1:**
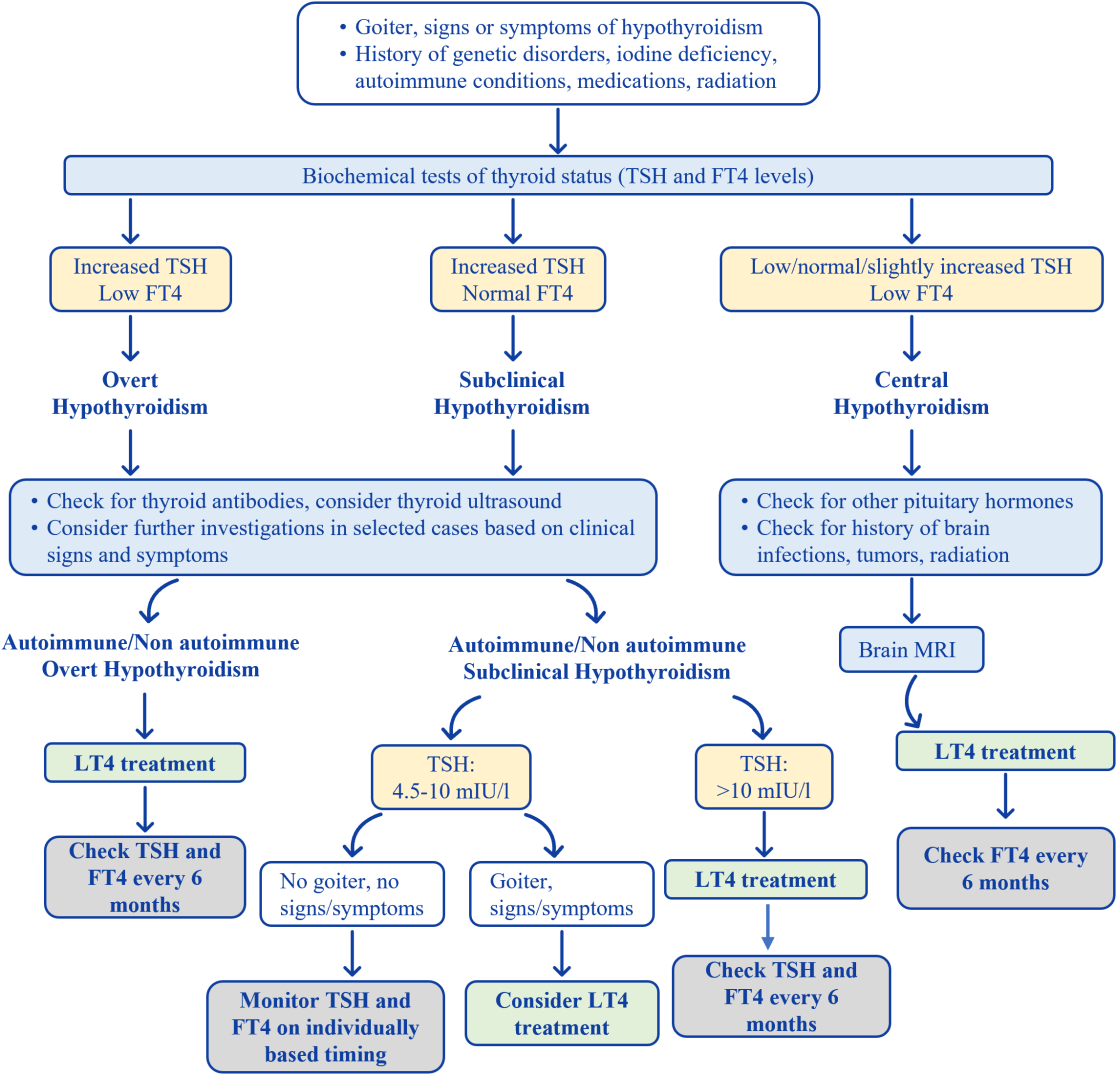
Overview of the diagnostic process and management of acquired hypothyroidism in children and adolescents.

A timely diagnosis of hypothyroidism in children is extremely important in order to avoid unfavorable consequences (8, 14). Increasing awareness of the typical and atypical signs or symptoms, particularly in children with a personal or family history of autoimmune diseases or thyroid disorders, may help in earlier recognition and treatment. **Figure 1** illustrates the proposed diagnostic approach and management strategy.

The initial approach to a pediatric patient may be prompted by various factors, including the presence of signs or symptoms suggestive of hypothyroidism, a family history of thyroid disease, an underlying chromosomal or autoimmune disorder, or the incidental detection of abnormal TSH or FT4 levels. Consequently, patient evaluation should include a comprehensive assessment of both personal and family history. It is essential to review potential risk factors that may affect thyroid function, including genetic or autoimmune disorders, iodine deficiency, medications, exposure to radiation and environmental disruptors, or obesity (8, 25, 60). This approach facilitates the identification of individuals at risk for thyroid disease, even in the absence of symptoms. Additionally, an accurate physical examination should be performed to identify the presence of goiter or other signs and symptoms of hypothyroidism (8).

Biochemical testing of TSH and FT4 levels remains the cornerstone for diagnosing hypothyroidism (8, 61). TSH is the most reliable marker for assessing thyroid status in most children, with abnormal circulating TSH levels being the earliest indicator of thyroid dysfunction. In contrast, FT3 has no role in the diagnosis of hypothyroidism, since its levels do not decrease until FT4 reaches critically low concentrations (8, 119). Generally, high TSH and low FT4 levels indicate overt primary hypothyroidism, while normal FT4 and slightly increased TSH levels indicate SH. For patients with SH, it is recommended to retest thyroid function after 4–12 weeks to determine whether the TSH elevation persists (4, 61). Low FT4 levels with inadequately normal or low TSH concentrations may suggest a diagnosis of acquired CeH (55). Moreover, in subjects with overt or SH, the evaluation of TPO-Ab and TG-Ab titers can differentiate children with autoimmune or non-autoimmune hypothyroidism. This is especially valuable as HT is the condition most often responsible for the onset of SH in childhood (8, 61).

In children with autoimmune hypothyroidism, thyroid ultrasonography completes the diagnostic evaluation. Indeed, a diffusely hypoechogenic and heterogeneous thyroid pattern is consistent with the biochemical diagnosis of HT (120), as observed for the case of patient 2. Additionally, the possibility of associated conditions or other autoimmune diseases should be carefully considered (8, 27).

When CeH is diagnosed, a comprehensive evaluation for additional pituitary hormone deficiencies is imperative, given the potential for panhypopituitarism. Neuroimaging, particularly magnetic resonance imaging (MRI), should be performed to detect structural lesions or abnormalities within the hypothalamic–pituitary axis. Early identification of these findings is crucial for guiding appropriate endocrine and neurosurgical management (55, 117).

Overall, further investigations should be assessed on a case-by-case basis, depending on the patient’s personal history, clinical manifestations, and neuroimaging and biochemical tests results.

Once acquired hypothyroidism has been confirmed or highly suspected, the patient should be promptly referred to a pediatric endocrinologist to ensure appropriate management and treatment. LT4 is the first-line treatment for managing hypothyroidism in children and adolescents (9). Its timely administration results in significant improvement in clinical symptoms and supports optimal recovery of growth and neurodevelopment, particularly in infants and young children, where thyroid hormone replacement is critical for intellectual and developmental outcomes (3). LT4 is administered orally, once daily, at least 30 minutes before breakfast, and the dosages vary according to the patient’s age, weight, and disease severity. In general, children require higher doses of LT4 per kilogram body mass to fully replace thyroid function compared to adults (12). While in children with congenital hypothyroidism, the recommended starting dose of LT4 is 10–15 mcg/kg/day, in children with acquired primary hypothyroidism, the suggested starting dose varies by age: 4–6 mcg/kg/day for children aged 1–3 years, 3–5 mcg/kg/day for those aged 3–10 years, and 2–4 mcg/kg/day for adolescents aged 10–16 years (3). However, the initial dose should be individualized. In cases of children with severe hypothyroidism, an initial low LT4 dose is recommended, progressively increasing over a few-week period, to avoid adverse events (60, 62). The treatment goal is to normalize TSH and FT4 levels in order to improve clinical manifestations and avoid adverse effects (13). In general, achieving a personalized treatment strategy is extremely important, as the individual optimal dosage can depend on several factors, including the etiology of hypothyroidism and the presence of conditions that can interfere with LT4 absorption (121).

Regular biochemical testing should be conducted. TSH and FT4 levels should be checked after 4–6 weeks following treatment initiation or dose adjustment to assess the dosage requirement (8). Once an optimal dose has been determined, the LT4 dosage should be monitored by assessing TSH and FT4 levels every six months until adult development is achieved (13).

In children with HT, a treatment with LT4 might also be considered in case of thyroid gland enlargement or nodular goiter (122–124), even in the absence of overt hypothyroidism. Overall, the benefits and potential effects of LT4 treatment must always be discussed with the family, and treatment should be continued only if it results in clear beneficial effects.

The management of children with SH remains controversial, as the condition is often transient and may resolve spontaneously, depending on the underlying etiology (61). LT4 treatment is recommended for subjects with elevated TSH levels (>10 mIU/l) or in the presence of clinical signs or symptoms of hypothyroidism. In children with only mildly elevated TSH levels (<10 mIU/l) and no clinical manifestations, a watchful waiting approach with regular monitoring is generally advised, although management should be individualized (4).

In children with HT, persistent SH is associated with a higher risk of progression to overt hypothyroidism compared to those with non-autoimmune SH (125). In case of non-autoimmune SH, management depends on the underlying etiology. For children with reversible etiologies, treatment should focus on modifiable factors and thyroid function should be reassessed after intervention.

Treatment with LT4 may be justified for patients with SH and a history of neck irradiation, in order to reduce the potential increased risk of thyroid cancer due to the trophic effect of TSH on thyroid epithelial cells, particularly in children with evidence of thyroid nodules (126). Furthermore, in children receiving medications known to interfere with thyroid function, such as amiodarone or antiepileptic drugs, it may be appropriate to initiate LT4 therapy for the duration of the pharmacological treatment. Thyroid function should be reassessed after discontinuation of the interfering medication to determine whether continued LT4 therapy is necessary. In children with idiopathic SH, LT4 therapy should be considered when TSH levels are above 10 mIU/L or in the presence of clinical symptoms; otherwise, careful monitoring is recommended (4).

In patients with CeH, LT4 therapy should only be initiated after adrenal insufficiency (AI) has been appropriately excluded. If AI is confirmed, glucocorticoid replacement must be commenced prior to LT4 administration to prevent the risk of adrenal crisis (54). LT4 treatment should be initiated with a low daily dosage of 25 μg, or even less for very young children, and gradually increased by 25 μg every 2–3 weeks until the full replacement dose is reached. The initial dosage may be adjusted based on the patient’s individual factors, including age and body weight (117). Monitoring of LT4 therapy should rely exclusively on FT4 levels, which should be maintained within age-appropriate reference ranges (54, 117).

In general, to accurately monitor dose adjustments, serum samples should be collected prior to the administration of the morning LT4 dose.

## Long-term outcomes and prognosis

LT4 therapy typically restores growth velocity (69, 127, 128); however, if diagnosis and treatment are delayed, final height may remain below average (65), as observed for patient 1. The severity of growth delay generally reflects the degree of hypothyroidism, with prepubertal children more likely to achieve normal adult height (66). Studies show that early treatment, particularly before puberty, results in better catch-up growth and more favorable height outcomes compared to treatment initiated during puberty (69, 70). Beyond growth, LT4 replacement has been shown to improve various other symptoms (91, 98, 107, 129), though evidence on its effects on cognitive dysfunction is limited. Improvements in academic performance and reduced impulsivity after treatment have been reported for children with acquired hypothyroidism; however, those with more severe disease had poorer baseline function and less post-treatment recovery (130).

Overall, the long-term outcome of acquired hypothyroidism depends on its underlying etiology. For instance, not all children with HT require lifelong LT4 therapy. A variable proportion of patients, between 16 and 25%, may experience spontaneous normalization of thyroid function and maintain a euthyroid state over time, with follow-up extending up to eight years (63, 131).

SH in children is usually benign and transient, with many cases resolving spontaneously without treatment (61). However, in those with HT, persistent SH is associated with a higher rate of progression to overt hypothyroidism (125), with over 50% developing overt hypothyroidism within five years (61). Furthermore, mildly elevated TSH levels in idiopathic SH are not generally associated with impaired growth or neurocognitive outcomes (4, 132). Indeed, two prospective studies found no significant benefit of two years of LT4 therapy on linear growth or cognitive function in children with idiopathic mild SH (133, 134). Whereas slight improvements have been observed in biochemical markers of cardiovascular risk and endothelial function following short-term LT4 treatment, although metabolic parameters remained within normal limits both at baseline and after therapy (133, 135).

## Back to the patients: practical considerations

Hypothyroidism in children can develop insidiously, and clinical and biochemical manifestations may remain unrecognized. The real-world cases described illustrate representative clinical scenarios that pose significant challenges to achieving a timely and accurate diagnosis of hypothyroidism.

The first case involved an unusual presentation of impaired renal function associated with typical manifestations such as short stature and pubertal delay, in the absence of goiter. This case highlights that unrecognized severe hypothyroidism during a critical growth period may compromise final adult height, despite normalization of renal function and growth velocity after treatment.

The second case illustrates how autoimmune hypothyroidism can manifest with rapidly worsening symptoms and multi-organ involvement, mimicking other metabolic conditions and significantly impacting the patient’s cognitive and overall health. However, despite the severity of hypothyroidism, no goiter was detected.

The third case is an emblematic example of CeH, highlighting how subtle symptoms can be, and underscores the importance of maintaining a high suspicion for CeH in the presence of reduced FT4 levels with normal TSH values. Prompt identification of CeH in this patient led to early diagnosis and treatment of craniopharyngioma, which had a favorable impact on prognosis.

The fourth case involved an uncommon and severe manifestation of hypothyroidism, primarily affecting the heart. For this patient, a timely and accurate diagnosis was crucial in preventing potentially life-threatening consequences.

As documented by these case reports, hypothyroidism can present with non-specific signs and symptoms, making differential diagnosis challenging. In severe cases, a missed or delayed diagnosis can result in deleterious consequences, emphasizing the need for clinical vigilance and a comprehensive diagnostic approach.

## Conclusion

Acquired hypothyroidism is a common endocrine disorder in the pediatric population, presenting with a broad spectrum of clinical signs and symptoms with varying severity. Atypical or subtle manifestations may complicate the differential diagnosis, potentially delaying recognition and treatment. These challenges underscore the importance of identifying both classic and less typical features of the condition. Early diagnosis and individualized management are essential for preventing serious complications and promoting optimal long-term growth and development in affected children. Indeed, most clinical manifestations are potentially reversible with levothyroxine therapy; however, growth can sometimes be definitively compromised, especially in severe chronic forms. Consequently, regular monitoring of thyroid function, growth parameters, pubertal development, and overall clinical outcomes in affected children is essential to maintain a stable euthyroid state and ensure a healthy transition into adulthood.
