# Correction: Unveiling the hidden: acquired pediatric hypothyroidism

**DOI:** 10.3389/fendo.2026.1797384

**Published:** 2026-02-10

**Authors:** Mariacarolina Salerno, Donatella Capalbo, Mariella Valenzise, Raffaella Di Mase, Letteria Anna Morabito, Chiara Centonze, Malgorzata Gabriela Wasniewska

**Affiliations:** 1 Department of Translational Medical Sciences, Pediatric Endocrinology Unit, University of Naples Federico II, Naples, Italy; 2 Department of Mother and Child, Pediatric Endocrinology Unit, University Hospital Federico II, Endo-ERN Center for Rare Endocrine Conditions, Naples, Italy; 3 Clinical Research Center DEMeTra, Department of Translational Medicine, University of Naples Federico II, Naples, Italy; 4 Department of Mother and Child, Pediatric Unit, University Hospital, Endo-ERN Center for Rare Endocrine Conditions, Messina, Italy; 5 Department of Human Pathology of Adulthood and Childhood, Pediatric Unit, University of Messina, Messina, Italy; 6 Medical Affairs, Merck Serono SpA, Rome, Italy

**Keywords:** acquired hypothyroidism, central hypothyroidism, Hashimoto thyroiditis, primary hypothyroidism, subclinical hypothyroidism

The **Conflict of interest** statement was erroneously given as

“Author CC was employed by company Merck Serono SpA.

The remaining author(s) declared that this work was conducted in the absence of any commercial or financial relationships that could be construed as a potential conflict of interest.

The reviewer AC declared a past co-authorship with the authors MS, RM, and MW to the handling editor.”

The correct **Conflict of interest** statement appears below.

“Author CC is employed by company Merck Serono SpA.

The remaining author(s) declared that this work was conducted in the absence of any commercial or financial relationships that could be construed as a potential conflict of interest.

The reviewer AC declared a past co-authorship with the authors MS, RM, and MW to the handling editor.

The authors MS, MV, MW declared that they were an editorial board member of Frontiers, at the time of submission. This had no impact on the peer review process and the final decision.”

The original version of this article has been updated.

